# Metazoan parasites of *Hoplias* aff. *malabaricus*, *Trachelyopterus galeatus* and *Schizodon borellii* (Osteichthyes) from the Protected Area and its main tributary, Brazil

**DOI:** 10.1590/S1984-29612023055

**Published:** 2023-10-23

**Authors:** Wagner Toshio Hasuike, Gabriela Michelan, Isabela Sales Quagliato, Heleno Brandão, Ricardo Massato Takemoto

**Affiliations:** 1 Programa de Pós-graduação em Biologia Comparada, Universidade Estadual de Maringá – UEM, Maringá, PR, Brasil; 2 Núcleo de Pesquisa em Limnologia Ictiologia e Aquicultura – NUPELIA, Universidade Estadual de Maringá – UEM, Maringá, PR, Brasil; 3 Programa de Pós-graduação em Recursos Naturais e Sustentabilidade, Universidade Tecnológica Federal do Paraná – UTFPR, Santa Helena, PR, Brasil

**Keywords:** Helminths, crustaceans, Actinopterygii, Atlantic Forest, São Francisco Falso River, Helmintos, crustáceos, Actinopterygii, Mata Atlântica, Rio São Francisco Falso

## Abstract

A study of the parasite-host interactions was conducted in the protected area popularly known as the “Refúgio Biológico de Santa Helena” and its tributary, the São Francisco Falso River. For each of the three host species, *Schizodon borellii*, *Hoplias* aff. *malabaricus*, and *Trachelyopterus galeatus*, 30 fish were collected from 2018 to 2019. A total of 2172 parasites were identified among the three host species. Among these, the Monogenea class had the highest number of species, with 26 taxa, followed by Copepoda with eight taxa, Digenea with six taxa, and Cestoda and Nematoda with one taxon each. Eleven new records of infection/infestation were found among the host species such as *Urocleidoides paradoxus*, *Urocleidoides ramentacuminatus*, *Rhinoxenus arietinus*, *Mymarothecioides* sp. (Monogenea), *Ergasilus* cf. *bryconis*, *Lernaea devastatrix*, and *Brasergasilus* sp. (Copepoda) parasitizing *S. borellii*. *Trinigyrus* sp., *Vancleaveus* sp. (Monogenea), *Gamispinus diabolicus* (Copepoda) present in *T. galeatus*, and *Gamidactylus jaraquensis* (Copepoda) present in *H*. aff. *malabaricus*. This study contributes to the record of parasite species occurrence in the vicinity of a protected area and helps fill gaps in the knowledge of fish parasitic fauna in the Neotropical region.

## Resumo

Foi realizado um estudo das interações parasitas-hospedeiros na área de proteção popularmente conhecida como “Refúgio Biológico de Santa Helena” e seu afluente, o Rio São Francisco Falso. Para cada uma das três espécies hospedeiras, *Schizodon borellii*, *Hoplias* aff. *malabaricus* e *Trachelyopterus galeatus*, foram coletados 30 peixes entre 2018 e 2019. Foi identificado um total de 2.172 parasitos entre as três espécies hospedeiras. Dentre estes, a classe Monogenea apresentou o maior número de espécies, com 26 táxons, seguida por Copepoda com oito táxons, Digenea com seis táxons, e Cestoda e Nematoda com um táxon cada. Foram encontrados onze novos registros de infecção/infestação entre as espécies hospedeiras tais como *Urocleidoides paradoxus*, *Urocleidoides ramentacuminatus*, *Rhinoxenus arietinus*, *Mymarothecioides* sp. (Monogenea), *Ergasilus* cf. *bryconis*, *Lernaea devastatrix* e *Brasergasilus* sp. (Copepoda) parasitando *S. borellii*. *Trinigyrus* sp., *Vancleaveus* sp. (Monogenea), *Gamispinus diabolicus* (Copepoda) presentes em *T*. *galeatus*, e *Gamidactylus jaraquensis* (Copepoda) em *H*. aff. *malabaricus*. Este estudo contribui para o registro da ocorrência de espécies de parasitos nas proximidades de uma área protegida, e ajuda a preencher lacunas no conhecimento da fauna parasitária de peixes na região Neotropical.

## Introduction

South America has the highest diversity of freshwater fish in the world (Reis et al., 2016; Birindelli & Sidlauskas, 2018). The orders Characiformes and Siluriformes have the highest species richness, with approximately four thousand valid species (Fricke et al., 2023). *Hoplias* aff. *malabaricus* Bloch, 1794, *Schizodon borellii* Boulenger, 1900 (Characiformes), and *Trachelyopterus galeatus* Linnaeus, 1766 (Siluriformes) are commonly found in the Paraná–Paraguay River basins, especially after the flooding of the Sete Quedas waterfall (Júlio et al., 2009; Reis et al., 2016; Ota et al., 2018; Reis et al., 2020).

Fish have been on Earth for a long time compared to other vertebrates and have provided more niches for invertebrates to conquer, being responsible for hosting more species of parasites than any other vertebrate group (Thatcher, 2006). Over the years, several authors have attempted to estimate parasite biodiversity and have estimated that one-third of the total species on Earth are parasites (Poulin & Morand, 2004; Luque et al., 2017).

Despite these uncertain estimates, efforts have been made in the form of catalogs on ictioparasitology in the Neotropical Region, including those on Monogenea (Cohen et al., 2013), Digenea (Kohn et al., 2007), Cestoda (Rego, 2000; Justo et al., 2017; Alves et al., 2017), Nematoda (Moravec, 1998; Luque et al., 2011), Acanthocephala (Santos et al., 2008), Isopoda (Thatcher, 2000), Copepoda (Luque et al., 2013), and all parasitological groups such as the study by Eiras et al. (2010). Notably, these host species, such as *S. borelli*, have previously been studied in other locations (Machado et al., 1996; Lacerda et al., 2007; Karling et al., 2011; Karling et al., 2014), and *T. galeatus* (Pavanelli & Santos, 1990; Kohn et al., 2011; Yamada et al., 2017; Yamada et al., 2021) and *H.* aff. *malabaricus*, which are considered the most studied hosts in various regions of Brazil (Gião et al., 2020; Lima et al., 2022; Diniz et al., 2022; Bueno et al., 2022; Duarte et al., 2023).

With increasing development of human activities, new challenges have emerged as mitigation measures for biodiversity conservation arising from changes in the biotic and abiotic environments (Kueffer & Kaiser-Bunbury, 2014). Areas created as environmental protection, is a way to keep intact this diversity of organisms, and transform unmanaged lands into well-managed entities that maintain this diversity (Rylands & Brandon, 2005). Conducting species surveys plays an important role in maintaining and understanding this biodiversity, assisting in a geographical analysis or changes in the composition of the biota (Wilson, 1988). With increasing research on global biodiversity in freshwater environments, it is becoming increasingly evident that parasites are a fundamental part of this diversity. Thus, they have been the least quantified group of organisms, despite playing fundamental roles as integral components of food webs and ecosystem functions, are significant in quantity and richness, therefore, they should not be neglected (Marcogliese, 2004; Thatcher, 2006; Lafferty et al., 2008; Timi & Poulin, 2020; Williams et al., 2022).

Considering the lack of ictioparasitological studies in the protected area and its main tributary, the São Francisco Falso River, this study aimed to provide the first record of parasite interactions with the hosts *H.* aff. *malabaricus, T. galeatus* and *S. borellii*. In addition to providing parasitological and ecological data on parasite diversity in this new study environment.

## Material and Methods

### Study area and host collection

The area of protection, referred to in the study region as the “Refúgio Biológico de Santa Helena”, was created in 1984 and has an area of 1,482.05 hectares (142.1 km^2^) and a perimeter of 30 km (Kliver, 2010). According to the same author, this ecosystem is located entirely in the municipality of Santa Helena, State of Paraná, and is isolated by an excavated canal with a concrete bridge and a gate for controlling the entry and exit of people, thus making it an artificial island.

The São Francisco Falso River was chosen to represent the area of influence of the aforementioned Conservation Unit owing to its important contribution to the formation of the Itaipu Reservoir, which is one of the largest floodplains in the municipality of Santa Helena, Paraná. This river has a watercourse of 127.04 km (Fronza, 2019), an area of 1,554 km^2^, a perimeter of 227.62 km, and covers the municipalities of Céu Azul, Diamante do Oeste, Matelândia, Ramilândia, Santa Helena, Santa Tereza do Oeste, São José das Palmeiras, São Pedro do Iguaçu, and Vera Cruz do Oeste (Lima et al., 2015).

**Figure 1:**
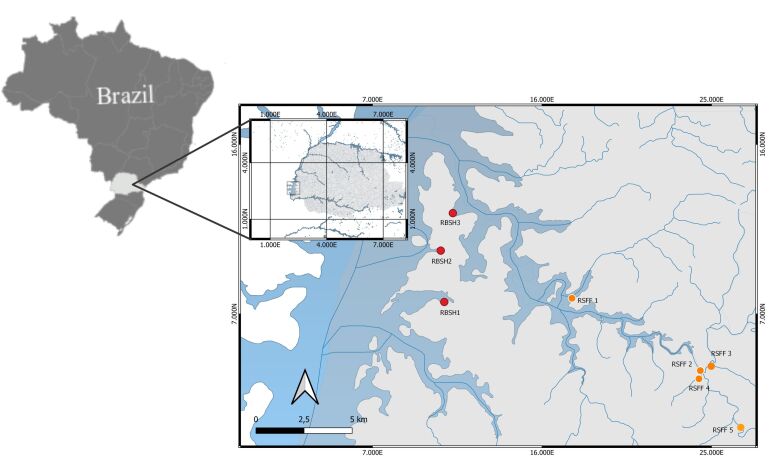
Brazilian map showing the sampling sites in the Refúgio Biológico de Santa Helena (RBSH) and Rio São Francisco Falso (RSFF), State of Paraná. (QGIS Geographic Information System. Open Source Geospatial Foundation Project).

The fish species studied were selected because they had the highest numerical representation within the collections. Thirty individuals of each fish species collected in the project were selected for parasitological studies. Sampling points were distributed around the “Refúgio Biológico de Santa Helena” (RBSH) along the course of the São Francisco Falso River (RSFF), totaling eight (8) sampling points (RBSH1: -24°51'15.12”S-54°21'21.12”W; RBSH2: 24°49'39.97”S-54°21'27.63”W; RBSH3: 24°48'30.50”S-54°21'5.33”W; RSFF1: 24°51'41.90”S-54°17'18.50”W; RSFF2: 24°53'14.14”S-54°13'6.60”W; RSFF3: 24°53'53.84” S-54°13'15.12”W; RSFF4: 24°53'18.56”S-54°13'30.32”W; RSFF5: 24°55'7.38”S-54°12'11.87”W) (Figure 1).

Representative specimens of the fish were deposited in the fish collections of Nupélia: *H.* aff. *malabaricus* (NUP:23044), *S. borelli* (NUP:23037), and *T. galeatus* (NUP:23107).

### Parasitological analysis

The following infection/infestation sites were analyzed: nasal cavities, gill filaments, intestine, eyes, heart, urinary bladder, and musculature. All host necropsy procedures, preservation, and parasite preparation were performed according to Eiras et al. (2006).

Representative specimens were deposited at the Helminthological Collection of the Oswaldo Cruz Institute (CHIOC) and the Helminthological Collection of the Institute of Biosciences at Unesp Botucatu (CHIB).

### Data analysis

To test the sufficiency of the samples, the species accumulation curve was calculated using the iNEXT package (Hsieh et al., 2016) was used. The ggplot2 package by (Wickham, 2016) was used to generate the parasite richness graphs. The ecological descriptors (Abundance, Prevalence, Mean abundance and Mean intensity) were calculated according to the method described by Bush et al. (1997). The parasitic diversity index was calculated using the Brillouin calculation (HB), and the Berger–Parker index (*d*) was used for parasitic dominance. All analyses were performed using the R software (R Core Team, 2020).

## Results

In the study, 40 parasite taxa and 2172 specimens were found, divided among the groups (Monogenea, Digenea, Cestoda, Nematoda, and Copepoda), present in the three host species analyzed in this study (see Tables 1, 2 and 3). All host individuals were parasitized by at least one parasite species.

**Table 1:** Total diversity of parasite species found in the host: *Schizodon borellii*.

**Parasite species**	**SI**	**A**	**P (%)**	**MA** ± SE	**MII** ± SE	**NIR**
**Monogenea**						
*Jainus piava* CHIOC- 39770	G	142	30.0	4.7 ± 16.8	15.7 ± 28.7	
*Urocleidoides paradoxus* CHIOC- 39779 a-d	G	24	16.6	0.8 ± 2.5	4.8 ± 4.8	X
*Urocleidoides ramentacuminatus* CHIOC-39780 a-d	G	14	30.0	0.4 ± 0.8	1.5 ± 0.7	X
*Rhinoxenus arietinus* CHIOC- 39772 a-d	NC	32	33.3	1.0 ± 2.6	3.2 ± 3.8	X
*Tereancistrum parvus* CHIOC- 39774	G	5	13.3	0.1 ± 0.4	1.25 ± 0.5	
*Tereancistrum paranaenses* CHIOC- 39773 a-c	G	3	10.0	0.1 ± 0.3	1.0	
*Mymarothecioides* sp.	G	7	3.3	0.2 ± 0.1	7.0	X
Dactylogyridae gen. sp.1	G	1	3.3	0.03 ± 0.1	1.0	
Dactylogyridae gen. sp.3	G	45	30.0	1.5 ± 2.7	5.0 ± 2.8	
**Digenea**						
*Austrodiplostomun compactum* (metacercariae) CHIOC- 39771	E	1	3.3	0.03 ± 0.1	1.0	
**Copepoda**						
*Gamispatulus schizodontis* CHIBB 702L-703L	NC	164	60.0	5.4 ± 8.8	9.1 ± 9.9	
*Ergasilus* cf. *bryconis*	G	16	20.0	0.5 ± 1.5	2.6 ± 2.7	X
*Lernaea devastatrix* CHIBB 693L; 694L e 695L	G	6	13.3	0.2 ± 0.5	1.5 ± 0.5	X
*Brasergasilus* sp.	NC	1	3.3	0.03 ± 0.1	1.0	X

SI: site of infection; G: gills; NC: nasal cavity; E: eyes; A: abundance; P (%): prevalence; MA: mean abundance; MII: mean intensity of infection; SE: standard error; NIR: new infection/infestation record.

**Table 2:** Total diversity of parasite species found in the host: *Trachelyopterus* *galeatus*.

**Monogenea**	**SI**	**A**	**P (%)**	**MA** ± SE	**MII** ± SE	**NIR**
*Cosmetocleithrum laciniantun* CHIOC-39764 a-d	G	622	90.0	20.7 ± 20.2	23 ± 20	
*Cosmetocleithrum baculum* CHIOC-39766 a-d	G	33	53.0	1.1 ± 1.5	2.0 ± 1.5	
*Cosmetocleithrum galeatum* CHIOC- 39767 a-c	G	68	66.6	2.2 ± 2.9	3.5 ± 3.0	
*Cosmetocleithrum spathulatum* CHIOC- 39768 a-c	G	318	93.0	10.6 ± 10.1	11.3 ± 10.1	
*Vancleaveus* sp.	G	2	6.6	0.06 ± 0.2	1.0	X
*Trinigyrus* sp.	G	1	3.3	0.03 ± 0.1	1.0	X
**Digenea**						
*Microrchis oligovitellum* CHIOC- 39765 a-b	I	24	50.0	0.8 ± 0.9	1.6 ± 0.6	
**Cestoda**						
*Cangatiella arandasi* CHIOC-39769	I	3	10.0	0.1 ± 0.3	1.0	
**Copepoda**						
*Gamispinus diabolicus* CHIBB 704L-705L -706L	NC	33	40.0	1.1 ± 1.8	2.7 ± 2.0	X

SI: site of infection; G: gills; NC: nasal cavity; I: intestine; A: abundance; P (%): prevalence; MA: mean abundance; MII: mean intensity of infection; SE: standard error; NIR: new infection/infestation record.

**Table 3:** Total diversity of parasite species found in the host: *Hoplias* aff. *malabaricus*.

**Monogenea**	**SI**	**A**	**P (%)**	**MA** ± **SE**	**MII** ± **SE**	**NIR**
*Anacanthorus* sp.1	G	2	6.6	0.06 ± 0.2	1.0	
*Anacanthorus* sp.2	G	1	3.3	0.03 ± 0.1	1.0	
*Anacanthorus* sp.3	G	4	3.3	0.13 ± 0.5	2.0 ± 1.4	
*Urocleidoides brasiliensis* CHIOC-39775 a-b	G	8	10.0	0.26 ± 0.9	2.6 ± 2.0	
*Urocleidoides cuiabai* CHIOC-39776 a-b	G	151	73.0	5 ± 5.1	6.8 ± 4.8	
*Urocleidoides paranae* CHIOC- 39778 a-d	G	35	13.0	1.16 ± 4.9	8.7 ± 12.2	
*Urocleidoides naris* CHIOC- 39777 a-f	NC	6	10.0	0.2 ± 0.7	2.0 ± 1.7	
*Urocleidoides* sp.1	G	82	70.0	2.8 ± 3.2	4.0 ± 3.2	
*Urocleidoides* sp.2	G	8	6.6	0.2 ± 1.2	4.0 ± 4.2	
Dactylogyridae gen.sp.1	G	15	13.0	0.5 ± 1.4	3.75 ± 1.5	
Dactylogyridae gen.sp.2	G	66	50.0	2.2 ± 3.0	4.4 ± 3.0	
**Digenea**						
*Austrodiplostomum compactum* (metacercariae) CHIOC-39783	E	9	20.0	0.9 ± 0.7	1.5 ± 0.8	
*Clinostomum dimorphum* (metacercariae)	H/I	7	20.0	0.2 ± 0.5	1.1 ± 0.4	
*Clinostomum* sp. (metacercariae) CHIOC -39784	I	2	6.6	0.06 ± 0.3	1.0	
*Phyllodistomum* sp. CHIOC- 39786 a-b	UB	8	10.0	0.2 ± 0.9	2.6 ± 2.0	
**Nematoda**						
*Contracaecum* sp. (larvae) CHIOC- 39785	I	10	13.0	0.2 ± 1.2	1.1 ± 3.0	
**Copepoda**						
*Gamidactylus jaraquensis* CHIBB 698L - 699L	NC	43	53.0	1.4 ± 1.8	2.68 ± 1.8	X
*Gamispatulus schizodontis* CHIBB 700L - 701L	NC	37	33.0	1.2 ± 2.7	3.7 ± 3.7	
*Lernaea devastatrix* CHIBB 696L e 697L	G	96	53.0	3.2 ± 5.1	6.0 ± 5.8	
*Lernaea devastatrix*	NC	7	20.0	0.2 ± 0.6	1.5 ± 0.8	

SI: site of infection; G: gills; H: heart; NC: nasal cavity; I: intestine; UB: urinary bladder; E: eyes; A: abundance; P (%): prevalence; MA: mean abundance; MII: mean intensity of infection; SE: standard error; NIR: new infection/infestation record.

**Figure 2:**
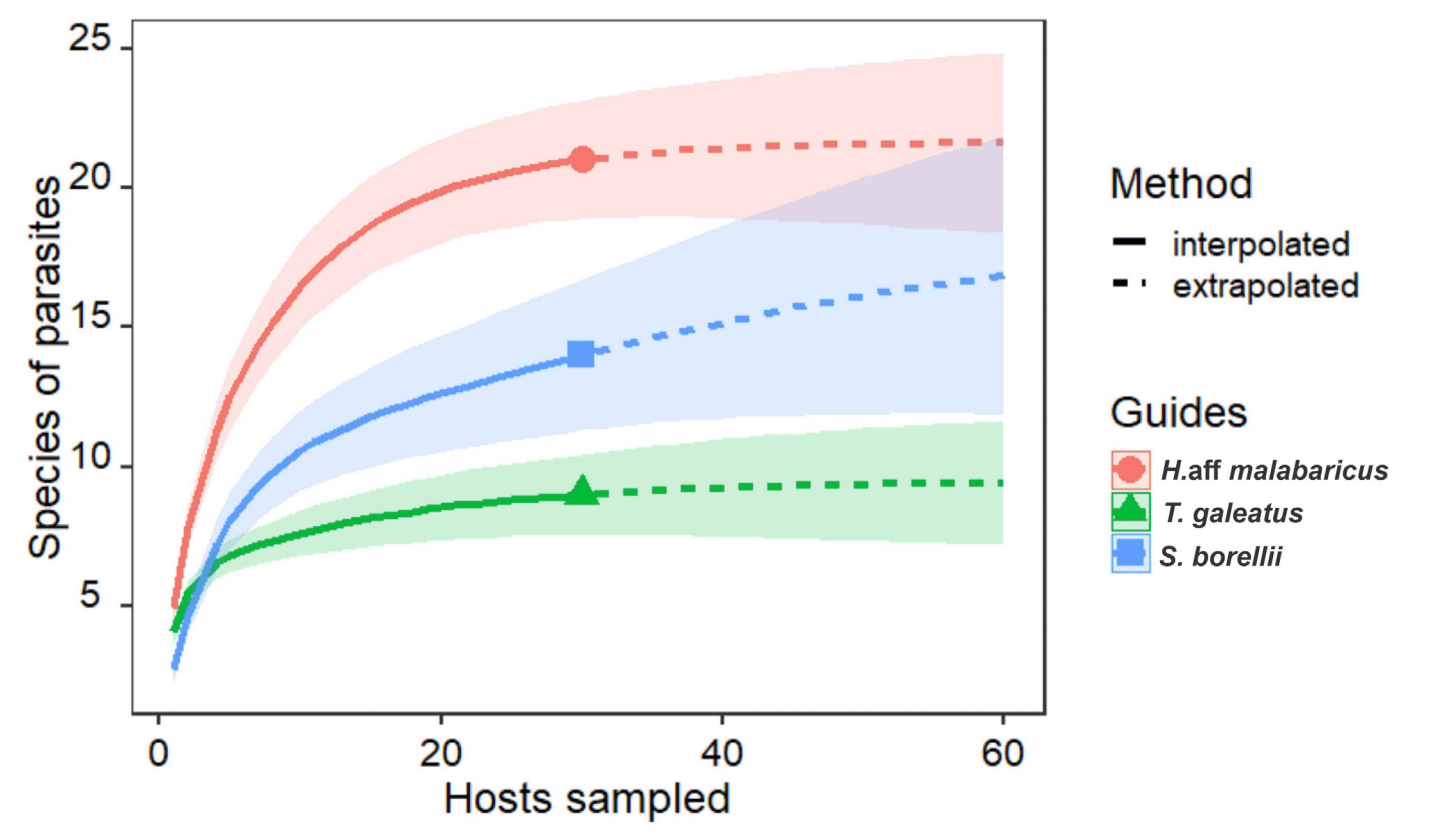
Accumulation curve of parasite species by hosts collected.

Although the available literature indicates that 30 individuals are adequate for parasitological studies, as shown in Figure 2, the species accumulation curve for *S. borellii* did not show stability, whereas the other two species reached stability with the amount collected.

**Figure 3:**
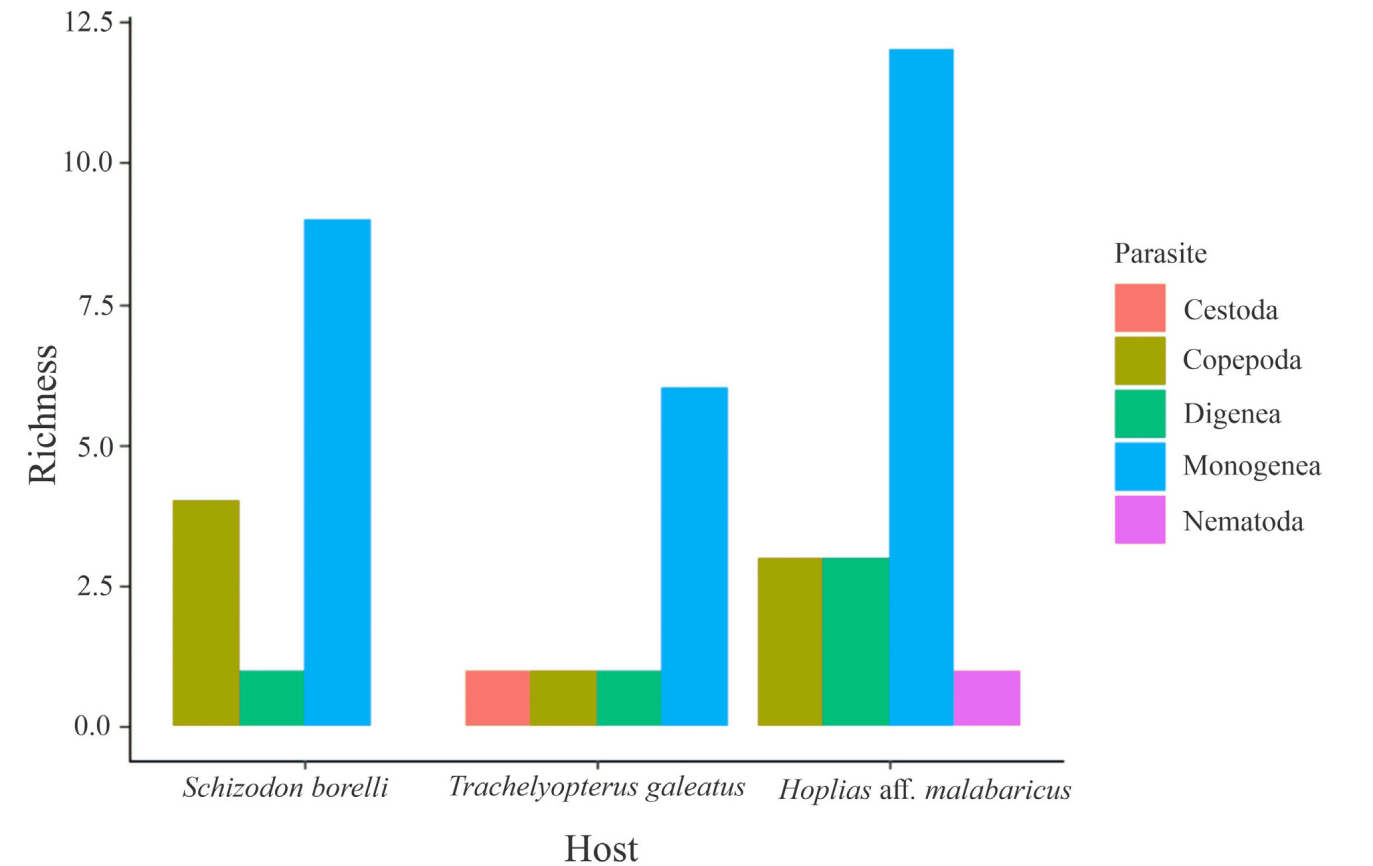
Total richness by parasite groups in each host species.

Among the host species, *H*. aff. *malabaricus* had the highest parasite richness, with 19 taxa, followed by *S*. *borellii* with 14 taxa, and *T. galeatus* with nine taxa (Figure 3).

The results obtained through the Brillouin index (HB) showed that *H*. aff. *malabaricus* had the highest diversity, followed by *T*. *galeatus* and *S*. *borellii.* The Berger-Parker index (*d*) showed that *T*. *galeatus* had the highest species dominance, followed by *S*. *borellii* and *H*. aff. *malabaricus* (Table 4).

**Table 4:** Brillouin index (HB) and Berger-Parker index (*d*), among the hosts species.

Host	Brillouin index (HB)	Berger-Parker index(*d*)
*Schizodon borellii*	0.45 ± 0.39	0.74 ± 1.22
*Trachelyopterus galeatus*	0.80 ± 0.25	2.41 ± 2.13
*Hoplias* aff. *malabaricus*	1.05 ± 0.39	0.37 ± 0.31

## Discussion

The Class Monogenea was the most expressive of the groups, with high parasite richness and abundance (61.9% of the parasites found), which may be related to some factors, the environment in which their hosts live, such as lakes or power plant reservoirs, which are characterized as lentic; thus, the specify, due their monoxenic life cycle, the free-swimming larvae can find their host more easily (Lizama et al., 2006). In addition, this group is considered to be the richest and most diverse among fish parasites which are highly specific to their hosts (Poulin & Morand, 2004; Kuchta et al., 2020).

Crustaceans were the second most diverse group in this study, accounting for 19% of the samples. Among the groups of metazoan parasites in freshwater fish, crustaceans Branchiura, Copepoda, and Isopoda stand out for their importance and diversity (Tavares-Dias et al., 2015). These three groups represent a large part of the parasitic crustacean fauna in the Neotropical region and deserve attention because they highly impact their hosts which are found mainly in fish farms or natural environments (Pavanelli et al., 2013). Ergasilids represent the fourth largest family of freshwater copepods, with over 60 species in the freshwater region of Brazil (Luque et al., 2013). Among those found in this study, *G. schizodontis* has a generalist habit, as it occurs in a variety of hosts and has been reported in four families: Anostomidae, Erythrinidae, Pimelodidae and Serrasalmidae (Narciso & Silva, 2020).

Digenea was the third most diverse group (14.3% of the samples found) and was the most recorded group in helminthological survey studies (Pavanelli et al., 1997; Takemoto et al., 2009; Lehun et al., 2020), as these parasites exploit fish as intermediate or definitive hosts in their life cycle. A parasitological survey conducted by Ramos et al. (2013) on infection and distribution of *Austrodiplostomum compactum* in Brazil, demonstrated that there is a wide range of fish species that are parasitized by *A*. *compactum*, encompassing the orders Characiformes, Perciformes, Siluriformes, and Gymnotiformes and, according to Yamada et al. (2008), the parasite presents a wide variety of intermediate hosts, thus presenting low specificity or “preference” to the various intermediate hosts already recorded.

In host fish, the diversity and richness of parasites are influenced by the set of species present in the environment, and most endoparasites are acquired by the trophic route, whereas the habitat, behavior, age, and sex of the hosts are important for ectoparasite infestation (Guidelli et al., 2003). The results obtained from the low prevalence and abundance of some species of endoparasites found in this study are interesting, and according to Overstreet (1997), the absence of parasites in a particular host indicates that the life cycle of the parasite is impaired. In this case, transmission between the mollusk (first intermediate host) and fish (second intermediate host) in the form of cercariae may not occur in this environment, making it impossible to close the cycle.

The Brillouin’s index (HB) calculation results showed two important findings. First, *S. borellii,* in which parasitic 14 taxa were found, showed a lower HB index than the *T. galeatus*, in which nine taxa were found (see Table 4). The difference in the HB index is because some species, such as Dactylogyridae gen. sp.1 (Monogenea), *A. compactum* (Digenea), and *Brasergasilus* sp. (Copepoda), occurred only once in the *S. borellii* sample and influenced the value of the diversity index. Second, it may be related to the number of hosts collected; as shown in Figure 2, the sample number of the host *S. borellii* was not stabilized in the species accumulation curve and probably did not demonstrate its real diversity. *Hoplias* aff. *malabaricus* was expected to have the highest parasite composition (19 taxa), mainly if we considered the behavior and trophic level of the host, as addressed by Poulin & Leung (2011).

Species such as *Vancleaveus* sp. and *Trinigyrus* sp. (Monogenea) present in *T. galeatus* and *Mymarothecioides* sp. (Monogenea) and *Brasergasilus* sp. (Copepoda) present in *S. borellii* had their first record of occurrence, but in low abundance and prevalence (below 10%). According to Bush et al. (1990), this type of case can be considered accidental if parasite indices are low. It is also important to highlight that this study is the first record of the occurrence of the remaining species: *Urocleidoides ramentacuminatus*, *U. paradoxus*, *Rhinoxenus arietinus* (Monogenea), *Ergasilus* cf. *bryconis*, *Gamispinus diabolicus*, *Gamidactylus jaraquensis* and *Lernaea devastatrix* (Copepoda). Thus, this study contributes to the knowledge of the occurrence of these parasitic species in fish of the families Anostomidae, Erythrinidae, and Auchenipteridae both in terms of their location and geographic distribution, providing new information for future studies on parasite diversity.

## References

[B001] Alves PV, de Chambrier A, Scholz T, Luque JL (2017). Annotated checklist of fish cestodes from South America. ZooKeys.

[B002] Birindelli JLO, Sidlauskas BL (2018). Preface: how far has neotropical ichthyology progressed in twenty years?. Neotrop Ichthyol.

[B003] Bueno RMR, Leite LAR, Pelegrini LS, Abdallah VD, Azevedo RK (2022). Biodiversity of the metazoan parasites of *Hoplias malabaricus* (Bloch, 1794) from the Jacaré-Pepira River, Tietê-Jacaré River Basin, São Paulo State, Brazil. Bol Inst Pesca.

[B004] Bush AO, Aho JM, Kennedy CR (1990). Ecological versus phylogenetic determinants of helminth parasite community richness. Evol Ecol.

[B005] Bush AO, Lafferty KD, Lotz JM, Shostak AW (1997). Parasitology meets ecology on its own terms: Margolis et al. revisited. J Parasitol.

[B006] Cohen SC, Justo MCN, Kohn A (2013). South American Monogenoidea parasites of fishes, amphibians and reptiles.

[B007] Diniz MFBG, Sousa WBB, Carvalho MNM, Yamada FH (2022). Metazoan parasite community of *Hoplias malabaricus* (Characiformes, Erythrinidae) in a stream of Caatinga domain, Brazil. Ann Parasitol.

[B008] Duarte R, Santos-Clapp MD, Brasil-Sato MC (2023). Metazoan endoparasites of *Hoplias malabaricus* (Bloch, 1794) (Actinopterygii: Erythrinidae) from upper and middle São Francisco river basin, Minas Gerais State, Brazil. Parasitol Res.

[B009] Eiras JC, Takemoto RM, Pavanelli GC, Adriano EA (2010). Diversidade dos parasitas de peixes de água doce do Brasil.

[B010] Eiras JC, Takemoto RM, Pavanelli GC (2006). Métodos de estudo e técnicas laboratoriais em parasitologia de peixes.

[B011] Fricke R, Eschmeyer WN, Van der Laan R (2023). Eschmeyer’s catalog of fishes: genera, species, references.

[B012] Fronza FL (2019). Determinação do potencial erosivo do solo nas bacias dos rios São Francisco Falso e São Francisco Verdadeiro, região oeste do estado do Paraná.

[B013] Gião T, Pelegrini LS, Azevedo RK, Abdallah VD (2020). Biodiversity of parasites found in the trahira, *Hoplias malabaricus* (Bloch, 1794), collected in the Batalha River, Tietê-Batalha drainage basin, SP, Brazil. An Acad Bras Cienc.

[B014] Guidelli GM, Isaac A, Takemoto RM, Pavanelli GC (2003). Endoparasite infracommunities of *Hemisorubim platyrhynchos* (Valenciennes, 1840) (Pisces: Pimelodidae) of the Baía River, Upper Paraná River floodplain, Brazil: specific composition and ecological aspects. Braz J Biol.

[B015] Hsieh TC, Ma KH, Chao A (2016). iNEXT: an R package for rarefaction and extrapolation of species diversity (Hill numbers). Methods Ecol Evol.

[B016] Júlio HF, Tós CD, Agostinho ÂA, Pavanelli CS (2009). A massive invasion of fish species after eliminating a natural barrier in the upper rio Paraná basin. Neotrop Ichthyol.

[B017] Justo MCN, Fernandes BMM, Knoff M, Cárdenas MQ, Cohen SC (2017). Checklist of Brazilian Cestoda. Neotrop Helminthol.

[B018] Karling LC, Bellay S, Takemoto RM, Pavanelli GC (2011). A new species of *Jainus* (Monogenea), gill parasite of *Schizodon borellii* (Characiformes, Anostomidae) from the upper Paraná river floodplain, Brazil. Acta Sci Biol Sci.

[B019] Karling LC, Lopes LPC, Takemoto RM, Pavanelli GC (2014). New species of *Tereancistrum* (Dactylogyridae) monogenean parasites of *Schizodon borellii* (Characiformes, Anostomidae) from Brazil, and emended diagnosis for *T. parvus*. Acta Sci Biol Sci.

[B020] Kliver SM (2010). Plano de manejo área de relevante interesse ecológico Santa Helena ARIE-SH Refúgio Biológico Santa Helena RBSH.

[B021] Kohn A, Fernandes BM, Cohen SC (2007). South American trematodes parasites of fishes.

[B022] Kohn A, Moravec F, Cohen SC, Canzi C, Takemoto RM, Fernandes BMM (2011). Helminths of freshwater fishes in the reservoir of the Hydroelectric Power Station of Itaipu, Paraná, Brazil. Check List.

[B023] Kuchta R, Řehulková E, Francová K, Scholz T, Morand S, Šimková A (2020). Diversity of monogeneans and tapeworms in cypriniform fishes across two continents. Int J Parasitol.

[B024] Kueffer C, Kaiser-Bunbury CN (2014). Reconciling conflicting perspectives for biodiversity conservation in the Anthropocene. Front Ecol Evol.

[B025] Lacerda ACF, Takemoto RM, Lizama MAP, Pavanelli GC (2007). Parasitic copepods in the nasal fossae of five fish species (Characiformes) from the upper Paraná River floodplain, Paraná, Brazil. Acta Sci Biol Sci.

[B026] Lafferty KD, Allesina S, Arim M, Briggs CJ, De Leo G, Dobson AP (2008). Parasites in food webs: the ultimate missing links. Ecol Lett.

[B027] Lehun AL, Hasuike WT, Silva JOS, Ciccheto JRM, Michelan G, Rodrigues AFC (2020). Checklist of parasites in fish from the upper Paraná River floodplain: an update. Rev Bras Parasitol Vet.

[B028] Lima FS, Melo HPS, Camargo LMA, Takemoto RM, Menguetti DUO, Virgilio LR (2022). Helminth parasites of *Hoplias malabaricus* (Bloch, 1794) in areas of Brazilian Amazon with different degree of deforestation. Conjecturas.

[B029] Lima VR, Fujita DS, Fujita RH (2015). Caracterização fluvio-morfométrica da Bacia Hidrográfica do Rio São Francisco Falso, estado do Paraná. Rev Norte Cient.

[B030] Lizama MAP, Takemoto RM, Pavanelli GC (2006). Influence of the seasonal and environmental patterns and host reproduction on the metazoan parasites of *Prochilodus lineatus*. Braz Arch Biol Technol.

[B031] Luque JL, Aguiar JC, Vieira FM, Gibson DI, Santos CP (2011). Checklist of Nematoda associated with the fishes of Brazil. Zootaxa.

[B032] Luque JL, Pereira FB, Alves PV, Oliva ME, Timi JT (2017). Helminth parasites of South American fishes: current status and characterization as a model for studies of biodiversity. J Helminthol.

[B033] Luque JL, Vieira FM, Takemoto RM, Pavanelli GC, Eiras JC (2013). Checklist of Crustacea parasitizing fishes from Brazil. Check List.

[B034] Machado MH, Pavanelli GC, Takemoto RM (1996). Structure and diversity of endoparasitic infracommunities and the trophic level of *Pseudoplatystoma corruscans* and *Schizodon borelli* (Osteichthyes) of the High Paraná River. Mem Inst Oswaldo Cruz.

[B035] Marcogliese DJ (2004). Parasites: small players with crucial roles in the ecological theater. EcoHealth.

[B036] Moravec F (1998). Nematodes of freshwater fishes of the Neotropical Region.

[B037] Narciso RB, Silva RJ (2020). Two *Gamispatulus* Thatcher & Boger, 1984 (Cyclopoida: Ergasilidae) from *Schizodon intermedius* Garavello & Britski (Actinopterygii: Anostomidae), with description of a new species. Zootaxa.

[B038] Ota RR, Deprá GC, Graça WJ, Pavanelli CS (2018). Peixes da planície de inundação do alto rio Paraná e áreas adjacentes: revised, annotated and updated. Neotrop Ichthyol.

[B039] Overstreet RM (1997). Parasitological data as monitors of environmental health. Parassitologia.

[B040] Pavanelli GC, Machado MH, Takemoto RM, Vazoller AESM, Agostinho AA, Hahn NS (1997). A planície de inundação do alto Rio Paraná: aspectos físicos, biológicos e socioeconômicos.

[B041] Pavanelli GC, Santos MHM (1990). *Cangatiella arandasi,* gen. n. sp. n (Cestoda-Proteocephalidae), parasito de *Parauchenipterus galeatus* (Siluriformes-Auchenipteridae) do Rio Paraná, PR. Rev Bras Zool.

[B042] Pavanelli GC, Takemoto RM, Eiras JC (2013). Parasitologia de peixes de água doce do Brasil.

[B043] Poulin R, Leung TLF (2011). Body size, trophic level, and the use of fish as transmission routes by parasites. Oecologia.

[B044] Poulin R, Morand S (2004). Parasite biodiversity.

[B045] Ramos IP, Franceschini L, Zago AC, Zica ÉOP, Wunderlich AC, Carvalho ED (2013). New host records and a checklist of fishes infected with *Austrodiplostomum compactum* (Digenea: Diplostomidae) in Brazil. Rev Bras Parasitol Vet.

[B046] Rego AA, Salgado-Madonado G, Aldrete ANG, Vidal-Martínez VM (2000). Metazoan parasites in the Neotropics: a systematic and ecological perspective.

[B047] Reis RB, Frota A, Deprá GC, Ota RR, Graça WJ (2020). Freshwater fishes from Paraná state, Brazil: an annotated list, with comments on biogeographic patterns, threats, and future perspectives. Zootaxa.

[B048] Reis RE, Albert JS, Di Dario F, Mincarone MM, Petry P, Rocha LA (2016). Fish biodiversity and conservation in South America. J Fish Biol.

[B049] R Core Team (2020). R: A language and environment for statistical computing. R Foundation for Statistical Computing.

[B050] Rylands AB, Brandon K (2005). Brazilian protected areas. Conserv Biol.

[B051] Santos CP, Gibson DI, Tavares LE, Luque JL (2008). Checklist of Acanthocephala associated with the fishes of Brazil. Zootaxa.

[B052] Takemoto RM, Pavanelli GC, Lizama MDLA, Lacerda ACF, Yamada FH, Moreira LHA (2009). Diversity of parasites of fish from the Upper Paraná River floodplain, Brazil. Braz J Biol.

[B053] Tavares-Dias M, Dias-Júnior MBF, Florentino AC, Silva LMA, Cunha AC (2015). Distribution pattern of crustacean ectoparasites of freshwater fish from Brazil. Rev Bras Parasitol Vet.

[B054] Thatcher VE (2006). Amazon fish parasites.

[B055] Thatcher VE, Salgado-Madonado G, Aldrete ANG, Vidal-Martínez VM (2000). Metazoan parasites in the Neotropics: a systematic and ecological perspective.

[B056] Timi JT, Poulin R (2020). Why ignoring parasites in fish ecology is a mistake. Int J Parasitol.

[B057] Wickham H (2016). Ggplot2: Elegant graphics for data analysis.

[B058] Williams MA, Faiad S, Claar DC, French B, Leslie KL, Oven E (2022). Life history mediates the association between parasite abundance and geographic features. J Anim Ecol.

[B059] Wilson EO (1988). Biodiversity.

[B060] Yamada FH, Moreira LHA, Ceschini TL, Takemoto RM, Pavanelli GC (2008). Novas ocorrências de metacercária de *Austrodiplostomum compactum* (Lutz, 1928) (Platyhelminthes: Digenea) parasito de olhos de peixes da bacia do rio Paraná. Rev Bras Parasitol Vet.

[B061] Yamada POF, Yamada FH, Silva RJ, Anjos LA (2017). A new species of *Cosmetocleithrum* (Monogenea, Dactylogyridae), a gill parasite of *Trachelyopterus galeatus* (Siluriformes, Auchenipteridae) from Brazil, with notes on the morphology of *Cosmetocleithrum striatuli.*. Comp Parasitol.

[B062] Yamada POF, Yamada FH, Silva RJ (2021). Three new species of *Cosmetocleithrum* (Monogenea: Dactylogyridae) gill parasites of *Trachelyopterus galeatus* (Siluriformes: Auchenipteridae) in Southeastern Brazil. Acta Parasitol.

